# Supplementary material for: Transcriptomic Analysis of Pubertal and Adult Virgin Mouse Mammary Epithelial and Stromal Cell Populations
Source: J Mammary Gland Biol Neoplasia. 2024 Jun 25;29(1):13. doi: 10.1007/s10911-024-09565-1 (PMC11199289; doi:10.1007/s10911-024-09565-1)
Supplement: Supplementary file 1 — Supplementary Material 1: Supplementary Fig. 1. Estrogen and progesterone fluctuations during the mouse estrous cycle. Supplementary Fig. 2. Estrous cycle monitoring of adult FVB/N mice and carmine staining of pubertal glands. Supplementary Fig. 3. FACS strategy. Supplementary Fig. 4. Estrous cycle monitoring of adult FVB/N mice for whole mount carmine staining. Supplementary Fig. 5. Whole mammary glands in estrus and diestrus show similar epithelial morphology. Supplementary Fig. 6. Unsupervised hierarchical clustering confirms proper isolation of the different cell populations. Supplementary Fig. 7 Estrous cycle dependent changes in cell proliferation genes. Supplementary Table 1. Differentially expressed genes with highest logFC in basal cells are directly linked to the cell cycle [84–89]. Supplementary Table 2. Differentially expressed Wnt genes during estrous cycle. [file 10911_2024_9565_MOESM1_ESM.pdf]

Supplementary Files

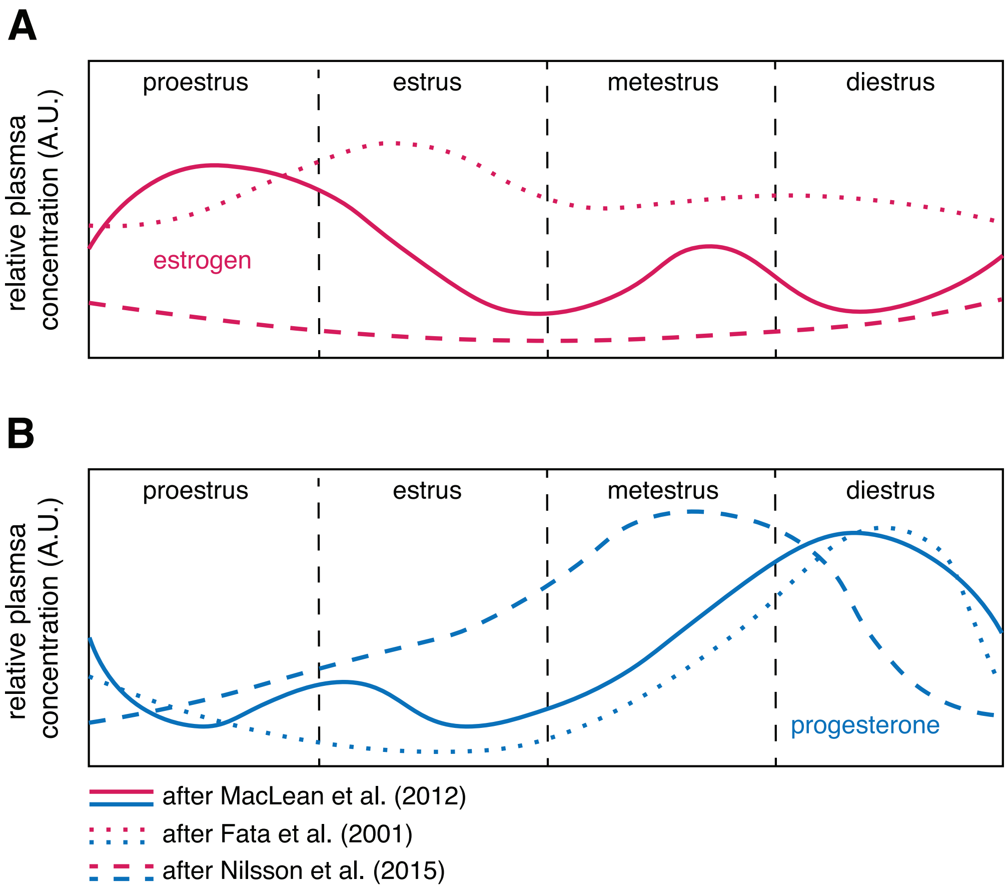

Supplementary Fig. 1. Estrogen and progesterone fluctuations during the mouse estrous cycle.

Cartoon depicting relative hormone plasma concentrations during the four stages of the mouse estrous cycle as reported in three different studies [13–15]. Source data available via https://osf.io/xv83g/ (SuppFig1_sourcedata.xlsx). A) The highest estrogen levels occur in proestrus/estrus. B) The highest progesterone levels occur in metestrus/diestrus. A.U. = arbitrary units.

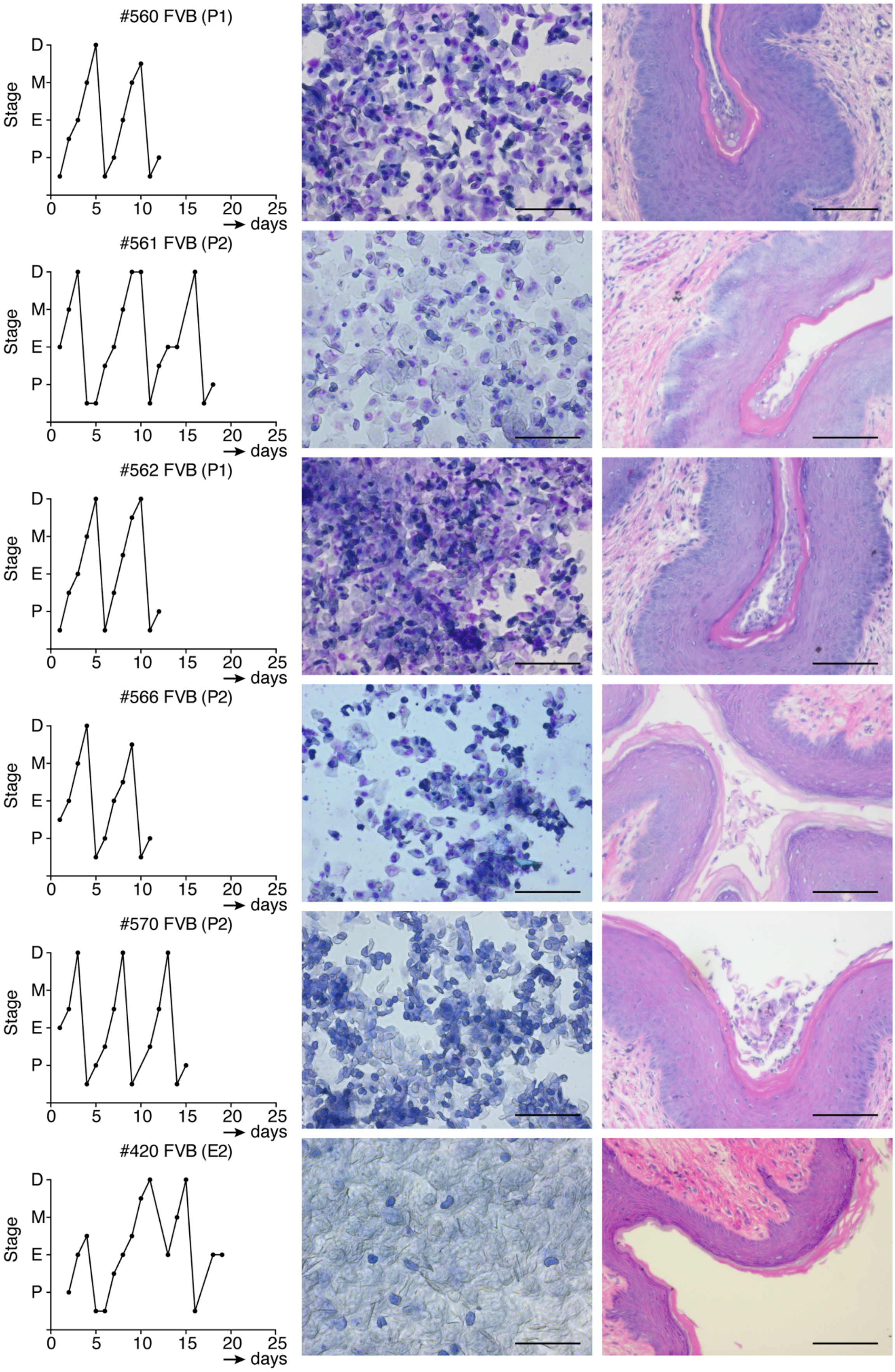
#560 FVB (P1)
#561 FVB (P2)
#562 FVB (P1)
#566 FVB (P2)
#570 FVB (P2)
#420 FVB (E2)
Stage
D
M
E
P
0 5 10 15 20 25
days

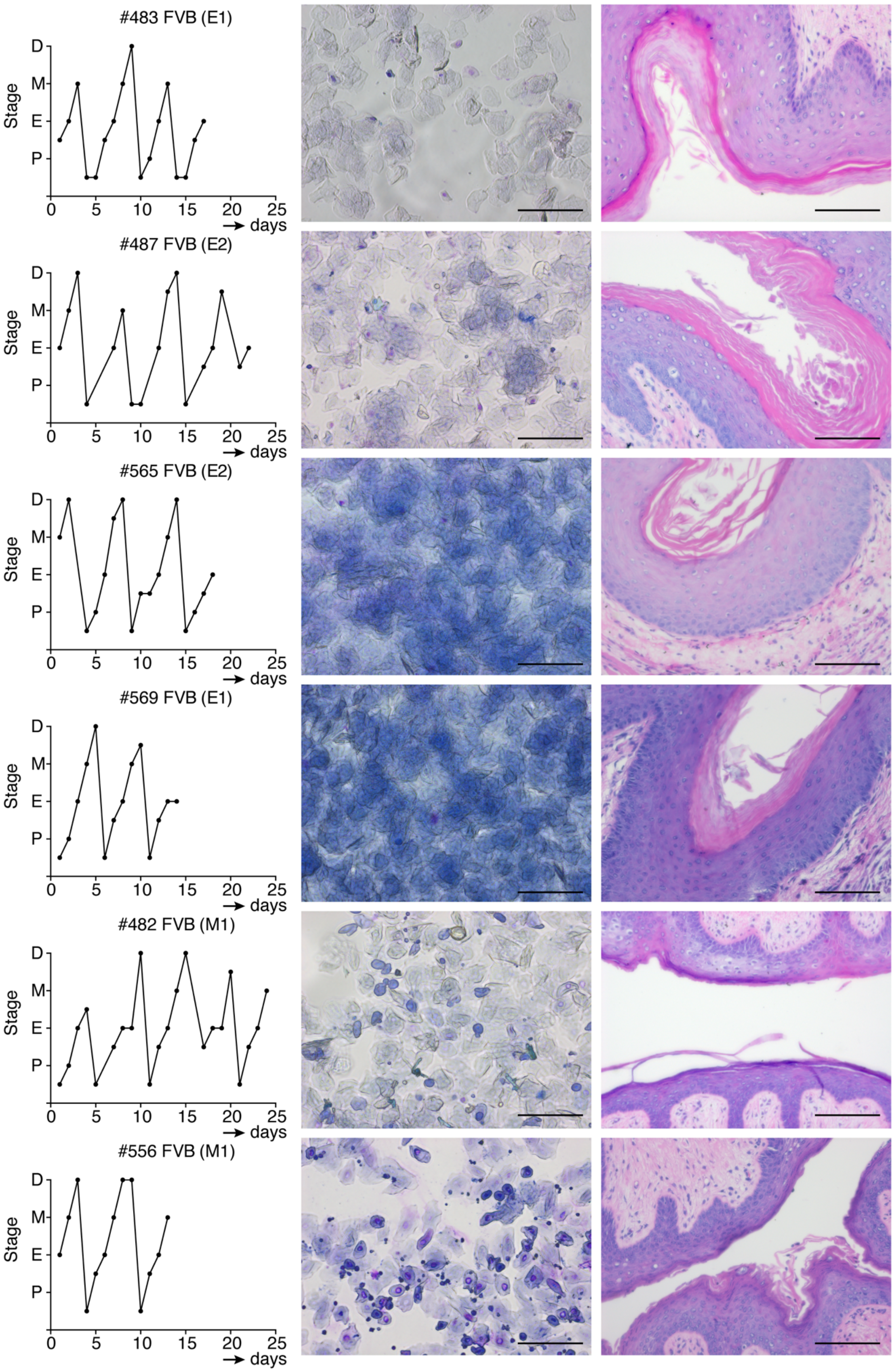
#483 FVB (E1)
#487 FVB (E2)
#565 FVB (E2)
#569 FVB (E1)
#482 FVB (M1)
#556 FVB (M1)
Stage
D
M
E
P
0 5 10 15 20 25
days

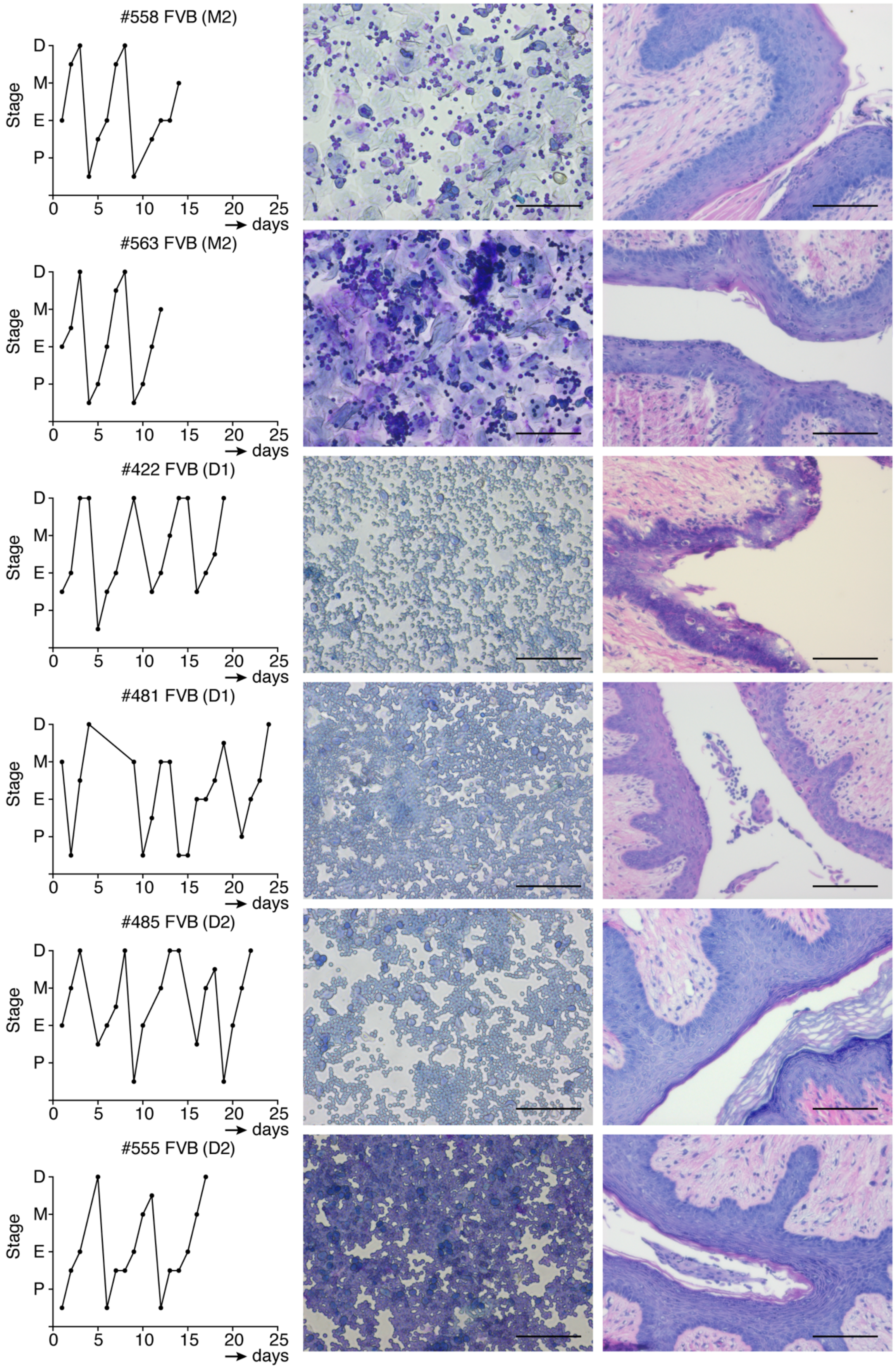
#558 FVB (M2)
#563 FVB (M2)
#422 FVB (D1)
#481 FVB (D1)
#485 FVB (D2)
#555 FVB (D2)
Stage
D
M
E
P
0
5
10
15
20
25
days

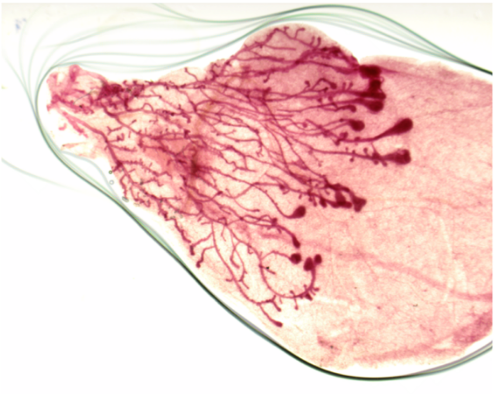

P36 example #3 mammary gland
(Carmine staining)

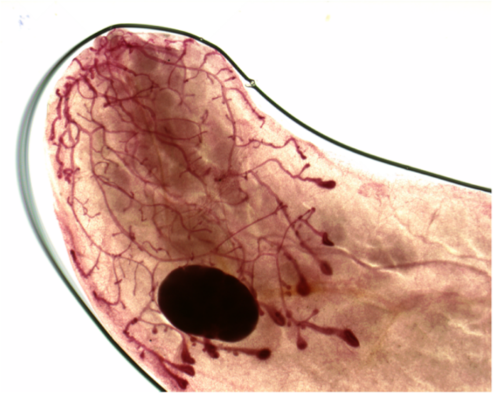

P36 example #4 mammary gland

Supplementary Fig. 2. Estrous cycle monitoring of adult FVB/N mice and carmine staining of pubertal glands. Rows 1-18: All mice that showed a stable estrous cycle and were selected for RNA-sequencing are shown in this figure, including the mice depicted in Fig.1. The left column shows estrous cycle monitoring over several days. The middle column and right column depict vaginal cytology and histology samples respectively, from the day of mammary gland isolation. Scale bar = 100 µm. Row19: Examples of pubertal mammary gland at P36, with the branching epithelium and terminal end buds (TEBs) at the tip visualized by carmine staining (not from one of the mice used for RNAseq).

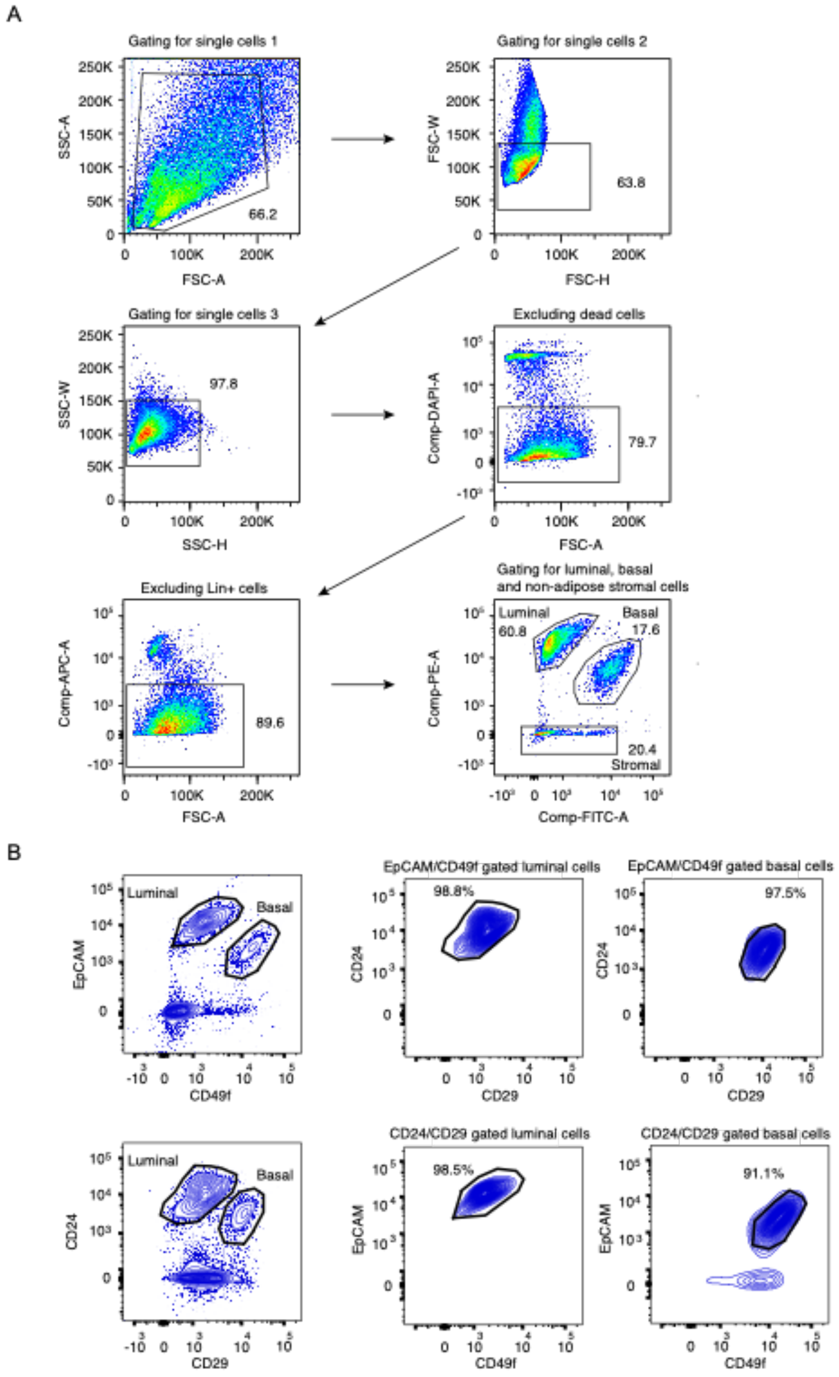

Supplementary Fig. 3. FACS strategy.

A) Gating strategy for sorting luminal, basal and non-adipose stromal mammary cell populations. SSC-A/FSC-A, FSC-W/FSC-H and SSC-W/SSC-H plots show gating for living single cells and to exclude dead cells, debris and doublets. DAPI plotted against FSC-A was also used to exclude dead cells. In the APC/FSC-A plot we gate for Lin- cells that are negative for CD45, CD31 and Ter119. CD45 and Ter119 markers were used to exclude haematopoietic cells and CD31 to exclude endothelial cells. EpCAM-PE and CD49f-FITC staining allowed us to gate for mammary luminal, basal and non-adipose stromal cells before sorting. B) Direct comparison of the CD24/CD29 and EpCAM/CD49f staining. In our hands, EpCAM/CD49f gives slightly better separation of basal and luminal populations, making it our staining method of choice.

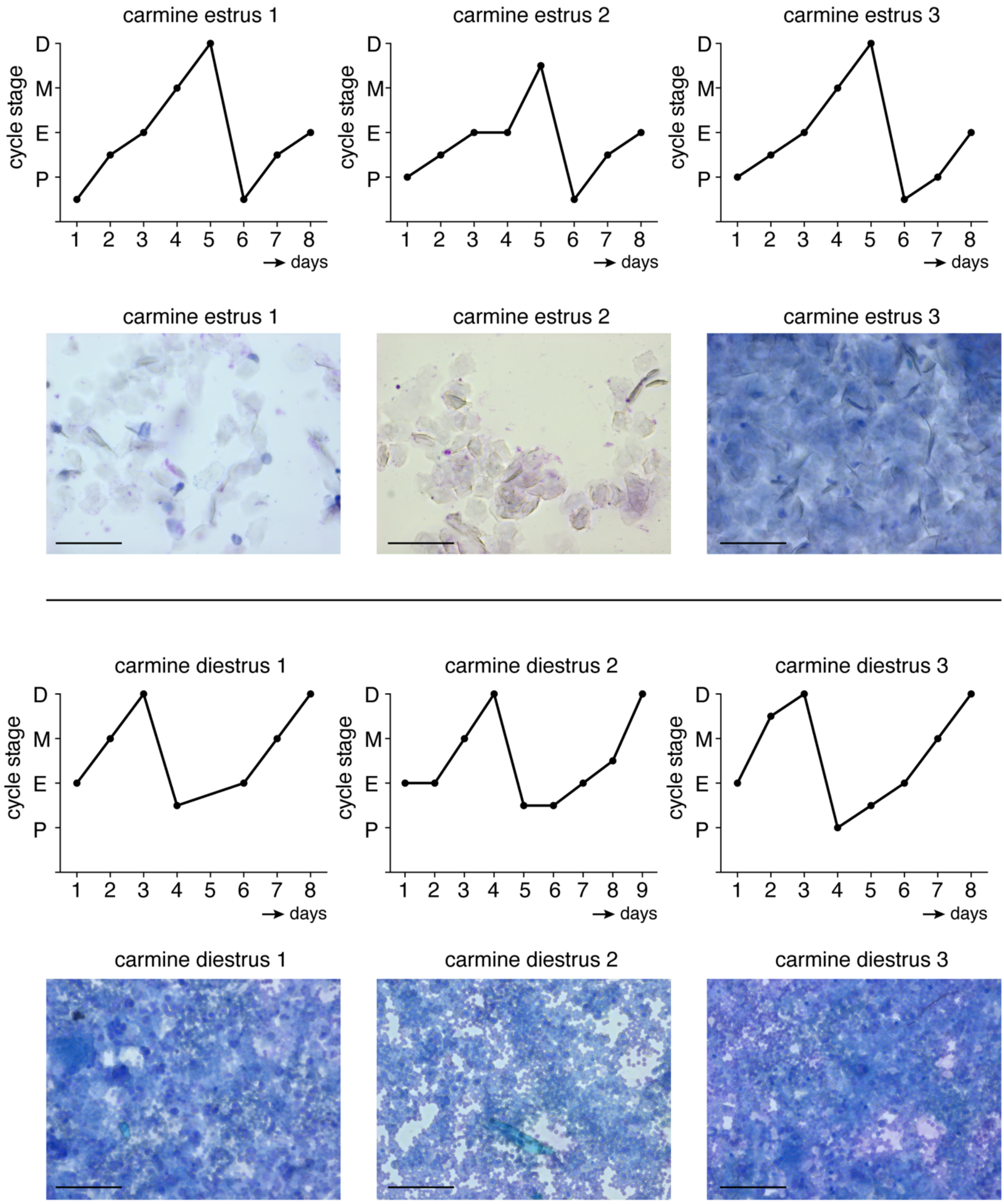

Supplementary Fig. 4. Estrous cycle monitoring of adult FVB/N mice for whole mount carmine staining.
N=6 stably cycling mice were selected for carmine staining, 3 in estrus and 3 in diestrus. This figure shows the graphs of monitoring the estrous cycle for at least one week using vaginal cytology. The shown image is the cytology sample on the day of mammary gland isolation. Scale bar = 100 μm. Samples 1, 2, and 3 correspond to the same numbers in Supplementary Figure 5.

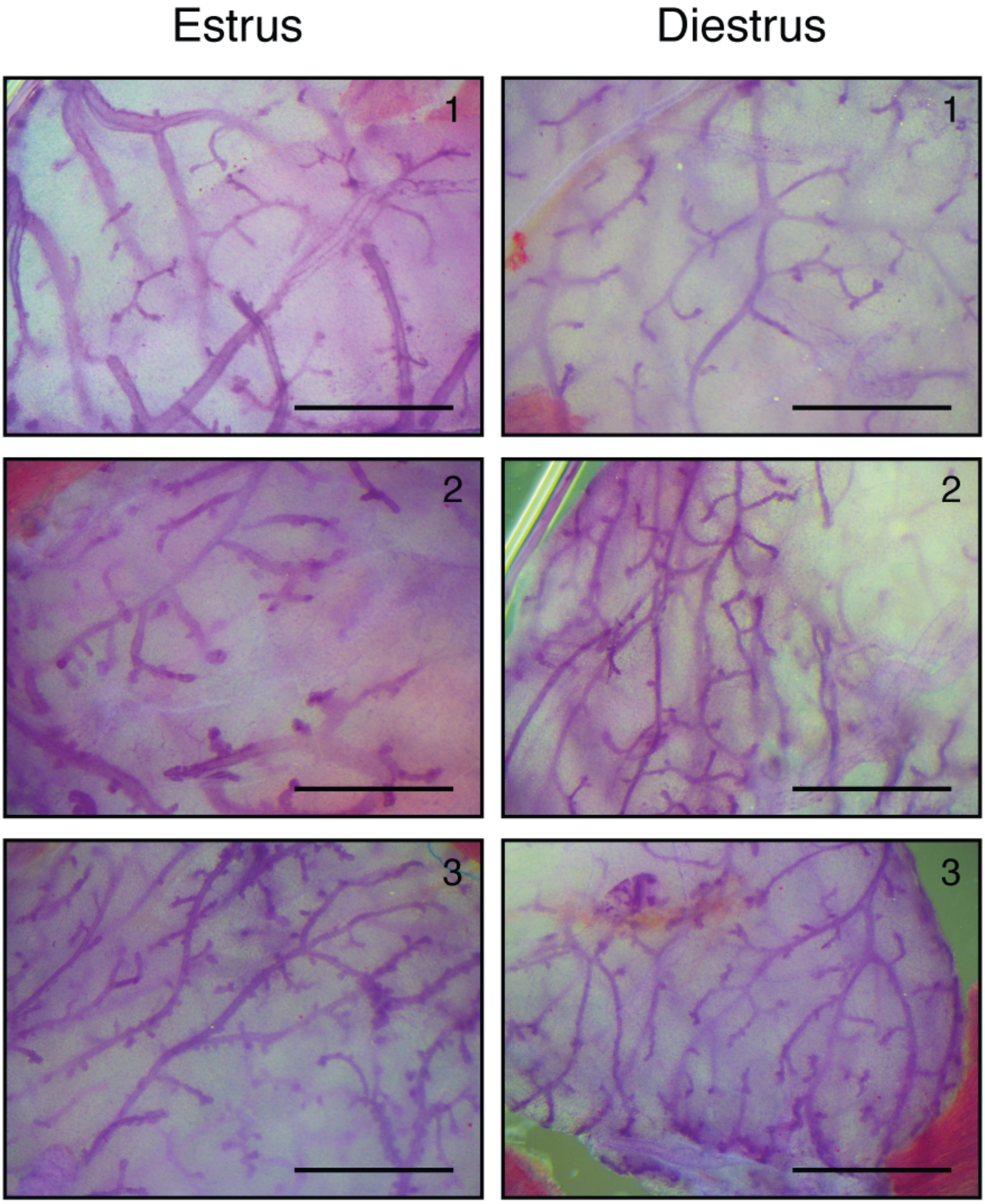

Supplementary Fig. 5. Whole mammary glands in estrus and diestrus show similar epithelial morphology. Images of carmine stained wholemount #3 mammary glands from n=6 different, estrous cycle staged mice (n=3 in estrus and n=3 in diestrus). Scale bar = 1 mm. Corresponding graphs of the estrous cycle monitoring of these 6 mice are provided in supplementary fig. 4.

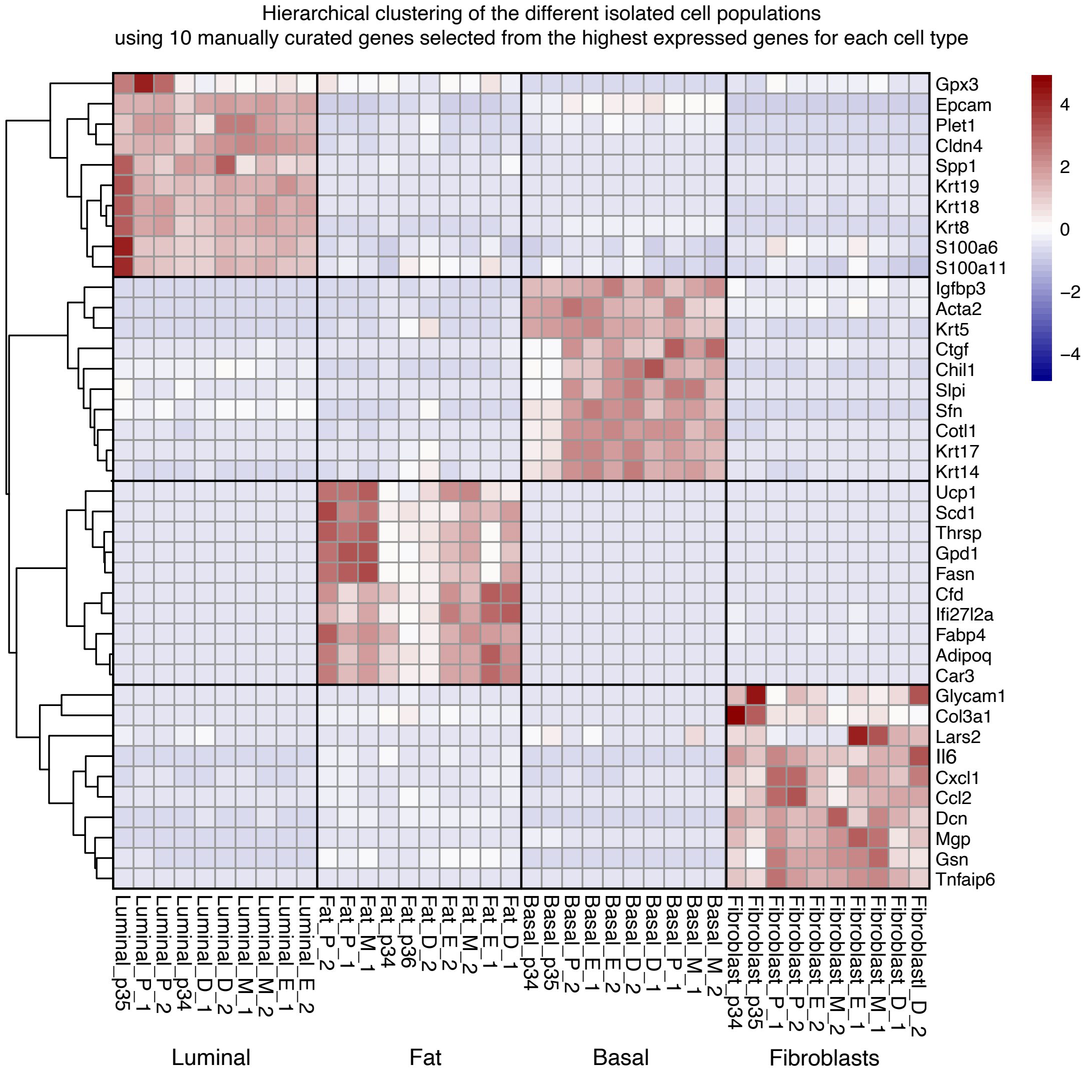

Supplementary Fig. 6. Unsupervised hierarchical clustering confirms proper isolation of the different cell populations. Heatmap showing expression (RPKM values, z-scale) of 40 genes (10 characteristic markers of each cell population) that were manually selected from the highest expressed genes across the different puberty and adult cell populations (top 35 for luminal, top 100 for basal, top 45 for fat and top 25 for non-adipose stromal). P, E, M and D: estrous cycle stages. 1 and 2: adult RNAseq replicates. p34, p35 and p36: puberty RNAseq replicates according to the samples in Table 1.

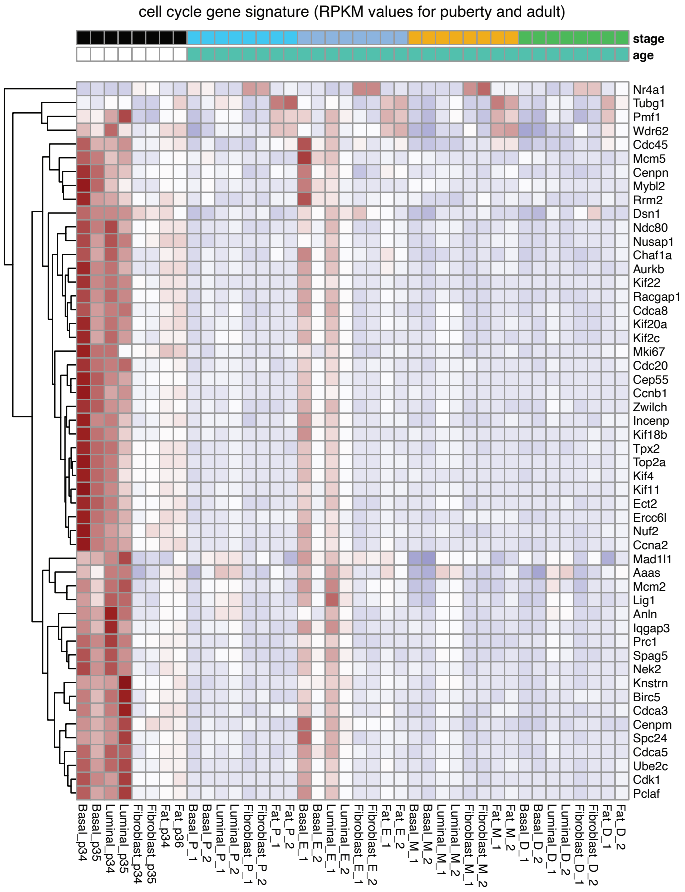

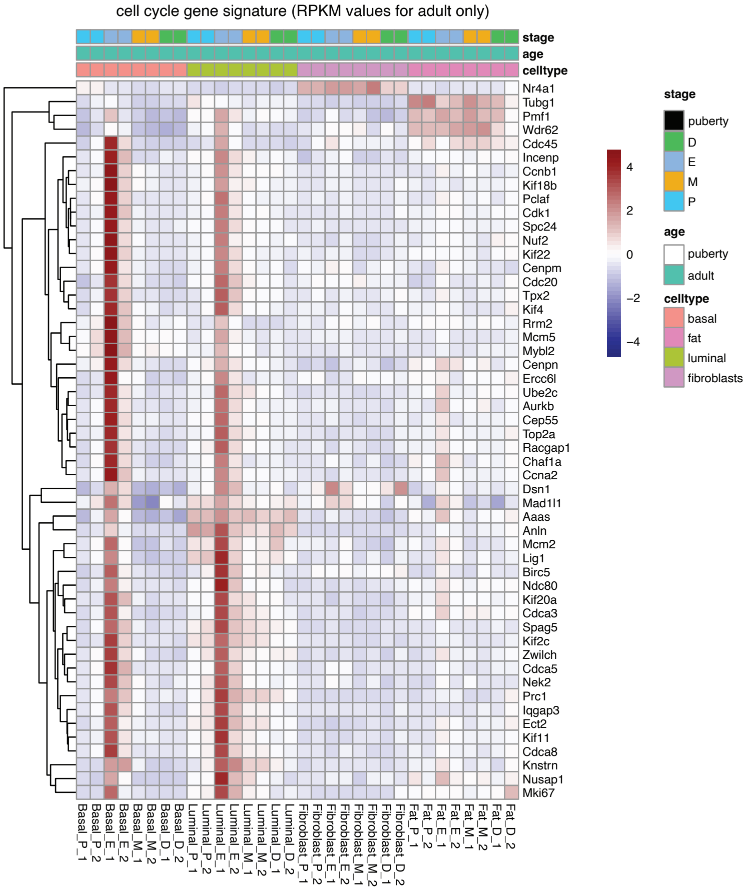

legend on next page

Supplementary Fig. 7 Estrous cycle dependent changes in cell proliferation genes.

Heatmap showing expression (RPKM values, z-scale) of cell division genes in epithelial, but not in non-epithelial cell populations. A total of 85 genes showed differential expression based on pairwise comparisons of different estrous cycle stages in basal cells (FDR<0.01, Fig. 5B). Of these 85 genes, 65 matched with the GO biological process *cell cycle* (https://www.gsea-msigdb.org/gsea/msigdb/mouse/geneset/GOBP_CELL_CYCLE.html). Expression of these 65 genes was plotted for each RNAseq replicate (1, 2) across the pubertal and adult populations (**a**) or across the different adult cell populations (**b**).

Supplementary Table 1. Differentially expressed genes with highest logFC in basal cells are directly linked to the cell cycle.

Gene	LogFC	LogCPM	FDR	Function	Ref
Iqgap3	-6.874	2.144	1.13E-03	Regulates cell proliferation through Ras/ERK pathway	[85]
Kif18b	-6.234	1.285	1.43E-03	Mediator of mitotic spindle stability	[86]
Ncapg	-6.183	1.453	3.25E-03	Subunit of condensing I complex, which regulates chromosome condensation and segregation during mitosis.	[87]
Pbk	-6.084	1.828	1.43E-03	Mitotic regulator, promotes cytokinesis, active during mitosis	[88]
Knl1	-5.102	1.099	5.24E-03	Plays a crucial role in spindle assembly checkpoint activation	[89]
Pclaf	-5.234	2.486	9.00E-03	PCNA-associated protein, essential for DNA replication and cell cycle progression	[90]

Genes with a LogFC of >|5| and LogCPM of >1 were selected from the total list of differentially expressed genes in basal cells, from all pairwise comparisons. These six genes fulfill those criteria. All six genes are upregulated in estrus compared to diestrus. LogFC (= log2 fold change) is the log-ratio of gene expression between diestrus and estrus. LogCPM (= log2 count per million) is the average expression taken over all samples within basal cells. FDR (= false discovery rate) is the expected proportion of false positive errors. We note that the normalized expression (CPM) of these genes is quite low and follow up research is required to determine which if any of these changes are biologically relevant.

Supplementary Table 2. Differentially expressed Wnt genes during estrous cycle.

Cell type	Comparison	***Wnt* gene**	logFC	logCPM	FDR
Adipocytes	D vs P	*Wnt7b*	3.724	5.529	4.90E-04
		Wnt5b	2.362	6.136	9.42E-04
		Wnt4	2.016	4.145	8.10E-03
		Wnt10a	3.490	3.167	1.20E-02
	D vs E	*Wnt5b*	2.268	6.136	1.39E-03
		Wnt7b	2.800	5.529	3.55E-03
	D vs M	*Wnt7b*	3.475	5.529	1.28E-03
		Wnt4	2.395	4.145	4.10E-03
		Wnt10a	3.486	3.167	1.78E-02
		Wnt5b	1.458	6.136	2.60E-02
Fibroblasts	D vs E	*Wnt6*	-1.630	1.450	4.42E-02
	D vs M	*Wnt5a*	-1.504	5.603	4.11E-02

Only in comparisons of adipocytes and non-adipose stromal cells differentially expressed (FDR <0.05) *Wnt* genes were present. Column "Comparison" shows the different pairwise stage comparisons that showed differentially expressed Wnt genes. P = proestrus, E = estrus, M = metestrus, D = diestrus. LogFC (= log2 fold change) is the log-ratio of Wnt expression between two different stages. LogCPM (= log2 count per million) is the average expression taken over all samples within a cell type. FDR (= false discovery rate) is the expected proportion of false positive errors. We note that the normalized expression (CPM) of these genes is quite low and follow up research is required to determine which if any of these changes are biologically relevant.
